# Semifluorinated Alkanes as New Drug Carriers—An Overview of Potential Medical and Clinical Applications

**DOI:** 10.3390/pharmaceutics15041211

**Published:** 2023-04-11

**Authors:** Charalambos Tsagogiorgas, Matthias Otto

**Affiliations:** 1 Department of Anaesthesiology and Critical Care Medicine, University Medical Center Mannheim, Medical Faculty Mannheim, University of Heidelberg, 68167 Mannheim, Germany; 2 Department of Anaesthesiology and Critical Care Medicine, St. Elisabethen-Krankenhaus, Teaching Hospital of the University of Frankfurt, 60487 Frankfurt, Germany

**Keywords:** drug carrier, drug delivery, excipients, fluorocarbons, medical application, semifluorinated alkanes

## Abstract

Fluorinated compounds have been used in clinical and biomedical applications for years. The newer class of semifluorinated alkanes (SFAs) has very interesting physicochemical properties including high gas solubility (e.g., for oxygen) and low surface tensions, such as the well-known perfluorocarbons (PFC). Due to their high propensity to assemble to interfaces, they can be used to formulate a variety of multiphase colloidal systems, including direct and reverse fluorocarbon emulsions, microbubbles and nanoemulsions, gels, dispersions, suspensions and aerosols. In addition, SFAs can dissolve lipophilic drugs and thus be used as new drug carriers or in new formulations. In vitreoretinal surgery and as eye drops, SFAs have become part of daily clinical practice. This review provides brief background information on the fluorinated compounds used in medicine and discusses the physicochemical properties and biocompatibility of SFAs. The clinically established use in vitreoretinal surgery and new developments in drug delivery as eye drops are described. The potential clinical applications for oxygen transport by SFAs as pure fluids into the lungs or as intravenous applications of SFA emulsions are presented. Finally, aspects of drug delivery with SFAs as topical, oral, intravenous (systemic) and pulmonary applications as well as protein delivery are covered. This manuscript provides an overview of the (potential) medical applications of semifluorinated alkanes. The databases of PubMed and Medline were searched until January 2023.

## 1. Introduction

Fluorinated compounds have been used in medicine and biomedical applications for decades because of their unique properties. Perfluorocarbons (PFCs) found their way into clinical practice, amongst others, as diagnostic tools [1,2] or in vitreoretinal surgery [2,3]. Their use as oxygen carriers in blood substitutes or liquid ventilation is an intriguing research field, yet has encountered many drawbacks and is still facing major hurdles [4]. Continuous advancements in PFC research, as reflected by the steady increase in the number of publications, are now paving the way for new medical applications, e.g., MRT, PET and nanoscale drug delivery systems [1,5,6,7].

**Figure 1:**
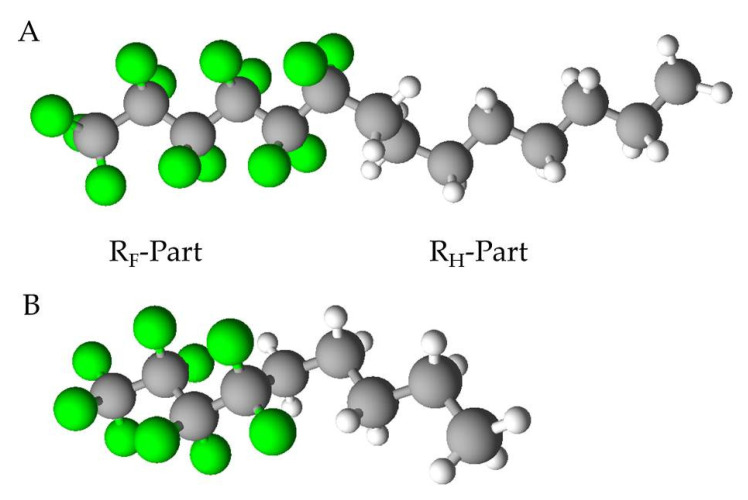
(**A**) Perfluorohexyloctane (F6H8) and (**B**) perfluorobutylpentane (F4H5) as example molecules for linear SFAs; R_F_-Part is the fluorinated part (green atoms), the R_H_-Part the hydrogenated (white) of the carbon chain (grey).

Semifluorinated alkanes (SFAs), are colorless non-aqueous liquids consisting of diblock molecules with perfluorocarbon (R_F_) and hydrocarbon (R_H_) parts with the chemical structure F(CF2)n(CH2)mH (see Figure 1). The nomenclature of SFAs is simplified FnHm, where n and m describe the number of carbon atoms in the fluorocarbon and hydrocarbon chains, respectively.

At first glance, the physicochemical properties of SFAs, as close relatives, resemble those of PFCs, e.g., due to strong intramolecular bonds, weak intermolecular interactions, high gas dissolving capacity and low surface tensions. SFAs are considered extremely hydrophobic, physically, chemically, physiologically inert, colorless, laser stable and, in comparison to PFCs, depict reduced densities—between 1.1 and 1.7 g/cm^3^ [8,9]. The oxygen solubility of 40 to 50 vol% (water: 3 vol%) also makes SFAs potential candidates for the synthesis of blood substitutes [8,10] and for liquid ventilation [11], but also potential candidates for organ preservation [12].

SFAs are already used clinically in ophthalmology for vitreoretinal surgery as endotamponades with silicone oil [13,14,15,16], but are also evaluated as new liquid eye drops for the administration of drugs dissolved or suspended in them [17,18,19,20].

Predominant features of these fluorinated amphiphiles are a tendency to self-aggregate into stable, well-organized supramolecular units such as vesicles and tubes [9,21]. Due to their high tendency to assemble at interfaces, they can be used to formulate a variety of multiphase colloidal systems, including direct and reverse fluorocarbon emulsions (e.g., with PFCs), microbubbles and nanoemulsions, gels, dispersions, suspensions and aerosols [9,21,22].

Unlike perfluorocarbons, SFAs have the potential to dissolve several lipophilic or water-insoluble substances and drugs, as suggested by Meinert et al. [23]. This makes SFAs potentially interesting as new excipients, especially as (co-) solvents or to be used as drug-delivery systems [21,23,24]. They were evaluated as drug carriers in liquid ventilation [11], as aerosol [25,26] or intravenously [27], but also for topical use in the eye [28] or on the skin [29,30].

The present work focuses, in particular, on the expanded range of potential medical applications [21,31] of these linear, liquid semi-fluorinated compounds (FnHm diblocks), and only briefly on their already clinically established use in ophthalmology [17,32]. The entire databases of PubMed, Medline and the website https://clinicaltrials.gov/ct2/home were accessed until 20 January 2023.

Section 1 provides brief background information on fluorinated compounds used in medicine and discusses SFAs, their physicochemical properties and biocompatibility. Section 2 focuses on their use in vitreoretinal surgery and new developments as drug-delivery eye drops. Section 3 discusses the oxygen transport of SFAs as pure liquids into the lungs and the intravenous application as SFA emulsions. Section 4 is dedicated to the drug-delivery aspects subdivided into topical, oral, intravenous (systemic) and pulmonary application as well as protein drug delivery.

## 2. Section 1—Physicochemical and Biological Aspects

### 2.1. (Poly-)Fluorinated Compounds

Polyfluorinated compounds do not occur naturally and have been produced for more than 50 years for a variety of industrial and household applications. They represent a large and complex group of organic substances with unique properties that are extremely versatile. Polyfluorinated chemicals are used in fluoropolymer-coated cookware, sportswear, military uniforms for extreme weather conditions, food processing equipment, medical devices, motor oil additives, fire extinguishing foams, paints and inks and water-repellent products [33]. The main characteristics of polyfluorinated compounds are the replacement of most hydrogen atoms with fluorine in the aliphatic chain. When all hydrogen atoms in aliphatic compounds (“hydrocarbons”) are replaced by fluorine, these organic fluorine compounds are called perfluorinated compounds and are better known as “perfluorocarbons” [34].

### 2.2. Fluorinated Compounds in Medicine

Fluorinated carbon chains confer unique biochemical properties to molecules and, due to their oxygen-transporting properties, may enable new therapeutic approaches, e.g., oxygen delivery (as blood substitutes) or liquid ventilation, but also may be used as new drug carriers, co-solvents or solubilizers [21].

**Figure 2:**
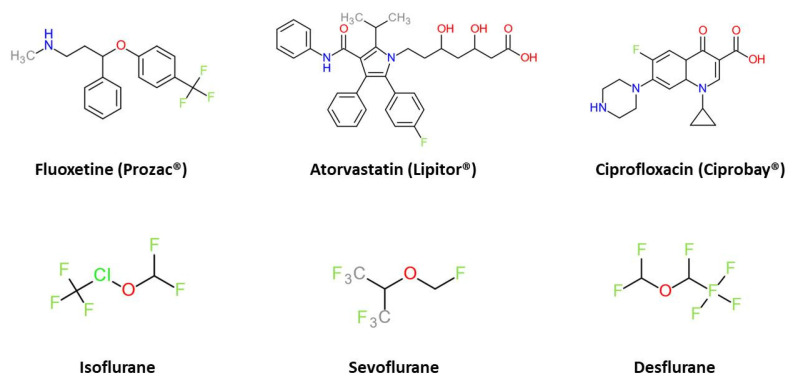
Important fluorinated compounds in medicine.

Numerous drugs, medications and delivery systems based on fluorinated substituents and compounds are in clinical use or under development. The use of fluorinated drugs and contrast agents is steadily increasing [35,36]. Amongst others, the top-selling fluorinated drugs include the antidepressant fluoxetine (Prozac^®^), the cholesterol-lowering drug atorvastatin (Lipitor^®^) and the antibacterial ciprofloxacin (Ciprobay^®^) [35]. Inhaled anesthetics are essentially fluorinated (e.g., isoflurane (CF_3_CHClOCHF_2_)), desflurane (CF_3_-CHFOCHF_2_), and sevoflurane ((CF_3_)_2_CHOCH_2_F)); as shown in Figure 2.

The isolated fluorine atoms or CF_3_ groups contribute to the binding affinity of a drug to the receptor, bioavailability, metabolism, half-life and pharmacokinetics, and to improve the efficacy/side-effect ratio [31]. A C_2_F_5_ group is present in the anticancer drug, fulvestrant (Faslodex^®^). C_2_F_5_-nitrosoimidazole is used for positron emission tomography (PET) as an effective PET radiopharmaceutical [37]. Microporous expanded PTFE (ePTFE or better known as GoreTex^®^) is used in reconstructive surgery, especially for intravascular bypass grafts. F-alkyl groups can modify the surface of polymers, including ePTFE, reduce thrombogenicity and friction, protect against enzymatic degradation and enhance the adhesion and growth of certain cells [38,39,40].

In brief, fluorine has found its way into biomedical applications for decades.

### 2.3. Physicochemical Properties of Semifluorinated Alkanes

The synthesis of semifluorinated alkanes (SFAs) was first reported in 1962 [41], but they immediately fell into oblivion. After more than 20 years, SFAs were rediscovered and characterized in 1984 and classified as a chemical link between two extreme families of compounds, n-alkanes and perfluorohydrocarbons, leading to very interesting and unusual properties [42].

SFAs (n-alkylalkanes) are diblock molecules with perfluorocarbon (R_F_ or “F-chain”) and hydrocarbon (R_H_ or “H-chain”) segments. The SFAs described here are “FnHm”-diblocks composed of linear perfluoroalkyl chains (F-chain, CnF2n+1) and linear perfhydroalkyl chains (H-chain, CmH2m+1), e.g., C_6_F_13_C_8_H_17_ (Perfluorohexyloctane; F6H8) [24,26].

SFAs are prepared by addition of a perfluoro-iodide to an n-alkene followed by dehalogenation with zinc powder and hydrochloric acid [43,44]. They exhibit a wide range of carbon chain lengths. SFAs with chain lengths up to 14 carbons are already used in medical applications [14,45], while compounds with 22 up to about 36 carbons are used in ski waxes [46]. The most prominent representative for medical applications is perfluorohexyloctane (F6H8), while for ski waxes it is FnH16, where n is an even number between 6 and 16.

SFAs are soluble in perfluorocarbon liquids (PFCL), hydrocarbons and silicone oils [23].

At first glance, the physicochemical properties of SFAs resemble those of their distant chemical relatives, the perfluorocarbons (PFCs), i.e., strong intramolecular bonds, weak intermolecular interactions, high gas dissolving capacity and low surface tensions (see Table 1). Similar to PFCs, these exceptional properties can be attributed to fluorine [31,47].

SFAs are considered extremely hydrophobic, thermally, chemically and laser stable as well as biologically inert [8,9,24]. Interestingly, the ratio between R_F_- and R_H_-segments determines their physicochemical properties [23,48].

**Figure 3:**
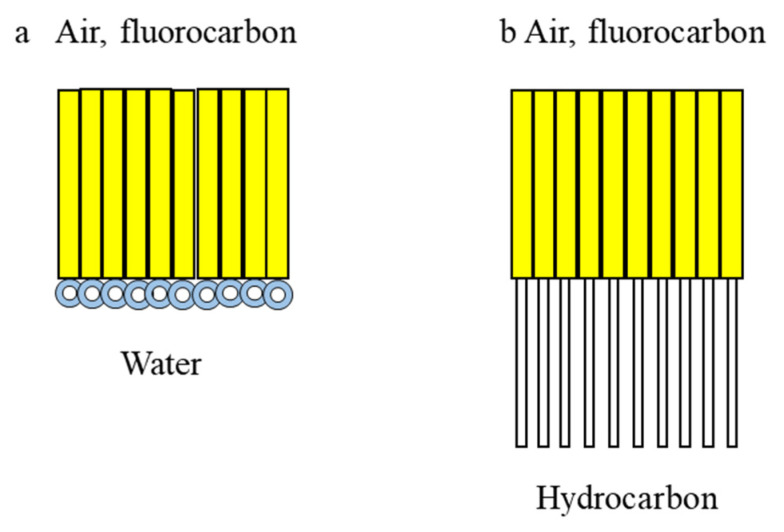
Fluorinated self-assembled films and colloids. (**a**) Monolayer of fluorosurfactant at the water/air (or water/fluorocarbon) interface; (**b**) illustrates the fact that F-alkyl and alkyl chains are mutually phobic: semifluorinated alkanes (CnF2n+1-CmH2m+1; SFA) organized as monolayers at hydrocarbon/air (or hydrocarbon/fluorocarbon) interface. Adapted from [6].

The linear FnHm diblocks have not only amphisteric, amphiphilic and amphidynamic properties but the chain conformations, cross sections and space requirements of the two chains differ significantly [24]. Compounds containing FnHm diblocks are valuable building blocks for self-assembling films and colloidal constructs [6,24,50]. The hydrophobicity plays an important role in self-assembly (e.g., for the formation of micelles, bilayer membranes, vesicles, fibers, etc.; see Figure 3) [51,52].

Interestingly, although many basic properties of these diblocks, such as density, surface tension and refractive index, are intermediate between those of their fluorinated or hydrogen-containing counterparts, important features such as the dielectric constant can be significantly different in some of the FnHm diblock compounds. In addition, some properties, e.g., a dipole moment and surface activity, are present in the diblock but not in the FC and HC counterparts (e.g., n-C_3_F_7_C_3_H_7_ versus n-C_6_F_14_ and n-C_6_H_14_) [24].

In brief, some of the physicochemical properties of the semifluorinated alkanes are unpredictable based on their F- or H-chain analogs.

### 2.4. Oxygen Solubility-Application as Oxygen Transport Carrier

The search for biocompatible O_2_/CO_2_ or NO carriers was not only conducted for PFCs but also extended to liquid SFAs [45]. The solubility of O_2_ is intermediate between that of the F- and H-chains of the same length. However, it should be noted that the solubility of CO_2_ in any solvent is usually ten-times greater than that of O_2_ [24].

Semifluorinated alkanes not only have low surface and interfacial tensions (about 45 and 19 mN/m, respectively) but also very high solubility for gases, such as oxygen and carbon dioxide. The solubilities of O_2_ in various liquid FnHm compounds and of two selected PFCs are listed in Table 2. The solubility of gases in SFAs is purely physical, with the larger the gas molecule, the better the solubility. Thus, gas release to tissue is kinetically uninhibited.

### 2.5. Semifluorinated Alkanes as Solvents

Due to their amphiphilic molecular structure, SFAs are hydrophobic but lipophilic. SFAs are amphiphilic substances with lipophobic RF and lipophilic RH segments. The unit of measurement for the lipophilicity of SFAs is the so-called CST value (critical temperature of solution). This indicates at which temperature a defined solvent mixture, consisting of the substance and a reference substance, shows a miscibility gap, which can be recognized by a separation of both substances. Table 1 lists the CST values of some selected SFAs in olive oil [49]. The lower the CST value the more lipophilic the SFA and the higher the potential solubility of lipophilic substances and drugs.

### 2.6. Semifluorinated Alkanes as Surfactants

As stated above, SFAs possess two moieties of opposite characters, with a perfluorinated chain and a hydrogenated fragment, and therefore are called “primitive surfactants” after Turberg and Brady [53,54].

The term “surfactant” in a traditional approach is referred to molecules possessing a hydrophobic “tail” and a hydrophilic “head”, which can be easily distinguished from each other. Surfactants are defined as diblock molecules, in which both moieties are of opposite character [53]. In such a molecule, both dislike moieties are covalently bound, but, if accounted separately, they would form two immiscible phases because of their reciprocal phobia. Scott and Hildebrand [55,56] reported for the first time the mutual immiscibility of fluorocarbons and hydrocarbons; interestingly, the phenomenon originates from the substantial difference between the atoms of fluorine and hydrogen. As already described for PFCs, the larger fluorine atoms with their higher electronegativity lead to larger cross-sectional areas in perfluorinated chains compared to hydrogen chains and form a dense electron shield around the carbon backbone [53,57].

Cohesive energy between perfluorinated chains is lower because of smaller polarizability of the fluorine atoms in comparison to hydrogenated chains. In consequence, the surface tension of perfluoroalkanes is lower (of about 10 mN/m) when compared to the equivalent hydrocarbons. Perfluorinated alkanes are much more hydrophobic than alkanes and, in addition, lipophobic. Covalent bonding of these highly incompatible moieties within one molecule results in the formation of semifluorinated alkanes (SFAs). Interestingly, perfluorinated chains adopt a twisted 15/7 helical conformation, whereas the analogous hydrogenated chain prefers a flat, all-trans chain arrangement [58,59].

The behavior of SFAs in the solid state, in solutions and monomolecular films is frequently surprising and contradictory to the common understanding of physical phenomena. From a physicochemical point of view, research with SFAs leads to a rethinking of paradigms.

In the solid state, for example, SFAs do not achieve one particular arrangement but crystallize in three complete different orderings, within which additional phase transitions occur [53]. It was also believed that liquid crystalline smectic phases are only formed by molecules with a rigid core substituted in the axial positions by some alkyl chains [53]. The work by Mahler et al. resulted in the abolishment of this belief, demonstrating that some SFAs are capable of smectic ordering [60]. Moreover, to be surface-active it was believed that amphiphilic molecules have to be composed of a hydrophobic tail and a hydrophilic head. This paradigm was abrogated, because it was shown for SFAs, as completely hydrophobic molecules, that they form micelles in hydrocarbon and fluorocarbon solutions [54]. Furthermore, they are capable to form Langmuir monolayers at the water/air interface [53,61]. The investigations of SFAs on Langmuir films are regarded as a breakthrough in this specific field of science changing general concepts and broadening the spectrum of available molecules to form those films [53].

### 2.7. Biocompatibility

Due to the lack of functional groups, SFAs are considered to be chemically as well as biologically inert. In addition, the compounds can withstand high-shear emulsion processing and do not seem to be affected by heat sterilization or high-energetic laser radiation [24]. When grafted onto a molecule, the F-chain of the SFAs seems to reduce hemolytic activity together with toxicity [62]. This, however, is not applicable to short compounds with branched F-alkyl groups which are not the subject of the present review, because they can display high inhalation toxicity [24].

Biocompatibility of SFAs seems to be dependent on their lipophilic behavior and, furthermore, on the molecular dimension of the semifluorinated alkanes used [63]. Increasing the length of alkyl chains leads to a higher prevalence of hydrocarbon properties, which inhibits the proliferation rate of human cell cultures. It was therefore supposed that SFAs with short perfluorinated alkyl chains and long alkyl (hydrocarbon) chains are also toxic [23]. Interestingly, pure SFAs from F10H2 to F6H10 showed to have no (toxic) influence on the proliferation on human carcinoma cell cultures (HELA and MonoMac cells) [23]. Acute toxicity in mice and rats was found to be very low. Extreme high lethal doses (LD50) of 2 g/kg bodyweight in rats for perfluorohexyloctane (F6H8) confirmed a high biocompatibility [23]. There has been no evidence of hemolysis or metabolism [24].

SFA adsorption, distribution, metabolism and excretion are still poorly understood as studies are lacking. After intravenous delivery of diblock emulsions given to rats, the excretion rate was shown to be dependent on dose, molecular weight and the F-chain-to-H-chain ratio. Most studies were conducted with double-bond diblock compounds (e.g., F6C=CHH10) [64], therefore the informative value for linear SFAs is limited. Nonetheless, there is a clear relationship with PFCs. A maximum concentration of these molecules was also observed in the liver and the spleen. Both organs are central part of the reticuloendothelial system (RES), which are responsible for clearing the blood of foreign particles [24]. Notably, the maximum concentration was found after day one and day seven. In summary, a diblock had a significantly shorter organ retention half-life than a non-lipophilic PCF of comparable size [24]. F6H8 is cleared from plasma within hours after a bolus injection, as shown by a sharp increase in signal in liver and spleen in NMR studies (unpublished observations as stated in [10]).

Biocompatibility in F6H8-containing emulsions (F6H8-cEM) seems to be dependent on the emulsifier used [65]. In a present study, two different F6H8-cEM were examined with either block copolymer Poloxamer 188 (Pluronic F68) or lecithin (S75) as the emulsifier and their impact on the RES and their inflammatory potential in human blood were evaluated. Pluronic negatively influences the biocompatibility of F6H8-cEM in vitro; phagocytosis is increased and therefore the F6H8-cEM with S75 in an animal model of acute kidney injury (AKI) was tested. No adverse effects on the damaged kidney were observed in vivo. Interestingly, the lecithin F6H8-cEM improved renal function compared to control. This study provides a scientific rationale for extended preclinical studies testing the use of lecithin-stabilized F6H8 emulsions as a carrier system for lipophilic agents during inflammatory processes without relevantly disturbing normal immune responses [65].

Moreover, a F6H8-containing emulsion used for propofol drug delivery was well tolerated after multiple intravenous injections in rats. Morphological examination of the H&E-stained sections by conventional light microscopy and overall histological assessment of the damage to the lung, kidney, liver and spleen after multiple F6H8-propofol emulsion administration revealed no morphological pathological changes and no significant differences between the groups [27].

## 3. Section 2—Clinical Use of SFAs in Ophthalmology

The use of PFC liquids in vitreoretinal surgery is part of clinical standard procedures. Because of its favorable proportions of RF- to RH-segments, the SFA perfluorhexyloctane (F6H8; see Figure 1A) was chosen for the first biomedical application in vitreoretinal surgery. After several months of internal tamponade, pure liquid F6H8, showing a specific gravity of 1.35 g cm^3^, was reported to be effective for the treatment of difficult retinal detachments without any evident side effects [14]. However, it has been reported that its use can lead to emulsification as well as the occurrence of other adverse reactions and, thus, poor long-term tolerance [66,67]. The use of F6H8 mixed with polydimethylsiloxane oils (also known as heavy silicon oils) reduced those side effects due to a much higher biocompatibility [68]. Over the past decade, they have been used as an intraocular tamponade during surgery for the treatment of retinal detachments [14]. Due to their higher solubility in silicon oils, SFAs have an advantage over PFCs [23]. A formulation of F6H8 and polysiloxane 5000 (Densiron 68) allowed successful long-term tamponade with complex retinal detachment with minimal side effects [69]. The first randomized prospective clinical trial to compare heavy and standard silicone oil in patients with proliferative vitreoretinopathy (PVR) of the lower retina (HSO study) failed to demonstrate superiority of a heavy tamponade [70].

The search for a biocompatible substance to achieve a better and easier silicone oil removal from the eye led to the use of perfluorobutylpentane (F4H5; see Figure 1B), which is a SFA that demonstrated excellent in vivo tolerance [63]. F4H5 was introduced as a new intraoperative agent in ophthalmology that allows a simple cleaning of silicone remnants from the retinal surface and, now, is clinically used as an effective wash-out agent after contact of silicone lenses with silicone oil [49]. According to Meinert et al. [23], F4H5 can be dissolved in silicone oil in any ratio. The small molecular size leads to a drastic reduction in the viscosity of the F4H5-silicone oil mixture and thus to a much more effective cleaning [49]. Moreover, F4H5 does not cause any inflammatory reactions after intraocular application [49]. No late complications were noted at 1-month follow-up, in particular, no retinal detachment was recorded [71]. The use of F4H5 in a randomized trial is described as safe and effective in reducing silicone oil residues [72].

A novel lipophilic tear supplement, containing 100% perfluorohexyloctane (F6H8; “EvoTears™”), has been introduced recently. Clinical studies have shown that F6H8 has a significant beneficial effect in patients with evaporative dry eye disease [19] and meibomian gland dysfunction [18] when administered topically for 6 to 8 weeks. Moreover, Carreira et al. evaluated the benefit of sodium hyaluronate (SH) versus SFA (F6H8) eye drops on tear film, meibomian glands and corneal epithelial thickness. F6H8-based eye drops significantly improved lipid layer thickness measurements in that study, which was at least responsible for restoring ocular surface homeostatic balance [32]. The authors concluded that smoking is associated with dry eye, meibomian gland dysfunction and corneal epithelial thinning that seem to be only partially reversible with topical lubricants, preferably an SFA [32].

Furthermore, a recently completed randomized phase 3 study (GOBI trial) in patients with dry eye disease (DED) associated with myoboma gland dysfunction (MGD) provides statistically significant and clinically meaningful evidence of a reduction in signs and symptoms of DED during eight weeks of treatment with F6H8 (NOV03). Perfluorohexyloctane (F6H8) was well tolerated in this patient group [73].

## 4. Section 3—Potential Bio-Medical Applications

### 4.1. SFAs as Oxygen Carriers

The topic of injectable oxygen delivery systems (“blood substitutes”) on the basis of fluorinated liquids has been extensively reviewed in the past [74]. Newer generations of PFC-based oxygen therapeutics were formulated with SFAs for better stabilization and particle size control. In those new formulations, F6H10 (equimolar to egg yolk phospholipids-(EYP)) for example was used to stabilize a perfluoroctylbromide (PFOB) emulsion. Interestingly, the amphiphilic F6H10 was used as “dowel” between the lipophilic EYP and the fluorophilic PFC perfluorooctylbromide (PFOB), which resulted in enhanced stability and O_2_-transport efficacy. This was because a high concentration of PFOB is now possible (up to 90% *w*/*v*) [75,76]. Several perfluorodecalin/SFA formulations were also studied, but with pluronic as emulsifier compared to the PFOB emulsions, resulting in lower stability [8,24]. Emulsions containing only the SFAs F6H10 and F10H2 as the dispersed phase were more stable than those prepared with perfluorodecalin, even when the latter was stabilized by adding diblocks [8]. Several studies showed stability and biocompatibility of F6H8-containing emulsions (F6H8-cEM) [10,27,65].

### 4.2. SFA-Containing Emulsions as Oxygen Carriers

In a rat model of transient focal brain ischemia, Seiffge et al. investigated the neuroprotective effects of enhancing the increase in blood oxygen-carrying capacity by applying an emulsion containing the SFA F6H8 in combination together with normobaric hyperoxygenation (NBO) [10]. They studied the spread of tissue hypoxia prior to filament-induced middle cerebral artery occlusion followed by a treatment of the rats with saline or with the F6H8-containing emulsion in two different doses (0.5 mL vs. 1.0 mL/100 g body weight).

The results of this study strongly support the concept that early tissue hypoxia plays an important role in the development of ischemic cell damage. The data also suggest that increased oxygenation by an artificial oxygen carrier in combination with NBO is superior to NBO alone. It may even be comparable to hyperbaric hyperoxygenation (HBO) by increasing arterial oxygen-carrying capacity without the need for sophisticated technical equipment such as a hyperbaric chamber. Indeed, the administration of an F6H8-containing emulsion in conjunction with oxygen breathing could be readily achieved in the prehospital setting [10]. Such a treatment regimen could not only have a significant neuroprotective effect, but also extend the time window for reperfusion therapy, as demonstrated in a previous experimental study with NBO [77].

Increasing the oxygen-carrying capacity of blood in the plasma compartment has a neuroprotective effect by mitigating the severity of hypoxia to the extent that it prevents cells from progressing to irreversible damage [10].

### 4.3. SFAs in Partial Liquid Ventilation

Perfluorocarbons have been used for both partial liquid ventilation (PLV) [78,79] and total liquid ventilation (TLV) [80,81,82]. In PLV, intrapulmonary instillation of perfluorocarbons in combination with conventional mechanical gas ventilation improves gas exchange in acute lung injury (ALI). TLV uses specially designed and developed ventilators that actively deliver a tidal volume of PFC to and from the lungs—so there are no air–liquid interfaces in this form of ventilation.

Dembinski et al. were the first research group to report PLV with SFA [11]. This study demonstrates the feasibility of PLV with the SFA perfluorohexyloctane (F6H8) in an (animal) experimental model of ALI and evaluates lung recruitment effects as a function of the SFA dose used. Furthermore, the authors investigated the efficacy of intrapulmonary drug deposition for the anti-inflammatory agents, α-tocopherol and ibuprofen. The authors conclude that SFA-PLV is feasible and results in pulmonary recruitment in ALI. Due to the lower density of SFAs, ranging from 1.2 to 1.7 g/mL, the risk of hemodynamic and circulatory effects during SFA-PLV is lower compared to PLV with PFCs. In addition, respiratory mechanics remained unchanged during SFA-PLV. The results of the study clearly demonstrate pulmonary lung recruitment caused by SFA-PLV, which is an important prerequisite for targeted drug delivery to previously atelectatic, non-ventilated lung areas. The efficacy of drug deposition in the lungs appears to depend on the distribution of drugs from the SFA phase into the blood.

### 4.4. Pancreas Storage in Perfluorohexyloctane (F6H8) for Islet Isolation

Pancreas shipment is frequently associated with prolonged ischemia deteriorating islet graft function. Using the PFC perfluorodecalin for human pancreas oxygenation is one way to prevent ischemic damage. Surprisingly, there is no improvement in isolation outcomes using this method [83]. Incubation of rat pancreas with the neat (non-emulsified) oxygenated SFA perfluorohexyloctane (F6H8) before islet isolation was found to be better compared to the use of perfluorodecalin [24,83]. Moreover, islet isolation performed after long-term pancreas preservation in humans can be significantly improved by utilizing F6H8 as oxygen carrier [12]. The same group published data which suggest that F6H8 does not increase islet yield but improves quality of pig islets isolated after prolonged cold ischemia [84]. Evaluation of oxygenated perfluorohexyloctane/polydimethylsiloxane (F6H8S5) for human pancreatic preservation (compared to usual preservation with University of Wisconsin solution or Custodiol) for clinical islet isolation and transplantation suggests a clear advantage of using F6H8S5 in human pancreases with prolonged cold ischaemia time, as it appears to allow prolonged cold ischemia time without compromising islet function and number [85].

### 4.5. SFA as Oxygen-Probes in 19F-MRI

To investigate the suitability of SFAs as reporter probes for 19F-oximetry, the longitudinal relaxation rate (R1) at a given oxygen partial pressure (pO_2_) and temperature (T) was investigated for three SFAs by Kegel et al. using a magnetic field of 9.4 T in vitro [86]. The SFA samples were investigated both as pure liquid (perfluorobutylpentane (F4H5), perfluoropentyloctane (F4H8) and perfluorohexyloctane (F6H8)) and for F6H8 as emulsion in vitro. The SFAs used in this study were very sensitive to pO_2_. Consistent with the behavior of other PFCs, the longitudinal relaxation rate of the three SFAs studied is a linear function of dissolved oxygen concentration and temperature. This linear relationship between R1 and pO_2_ for the 19F resonances of fluorinated molecules is well-known and has been confirmed for SFAs. Compared to other PFCs used in oximetry, the investigated SFAs show a higher sensitivity to temperature changes. F6H8 shows increased temperature sensitivity and could therefore be predestined for clinical use as a hypoxia marker as well as for temperature monitoring. The feasibility of 19F-oximetry with SFAs seems possible, especially for F6H8 as a pure liquid and emulsifier, which is a prerequisite for non-invasive oxygen tension measurements in vivo with 19F-MRI [86].

## 5. Section 4—Drug-Delivery Capability of SFAs

### 5.1. SFAs for Topical Drug Delivery on the Eye

Following the encouraging clinical results of topical application of SFAs to the eye, Gehlsen et al. used SFAs as drug carriers at the ocular surface [87]. Experimentally, cyclosporin A dissolved in F4H5 is equally effective compared to a commercial cyclosporin A formulation, but with a significantly faster therapeutic response in reducing signs of dry eye disease [87]. The SFA-based cyclosporine A formulation tested in a phase 2 clinical trial and a phase 2b/3 clinical trial showed significant improvements in corneal staining as early as two weeks after treatment initiation [88,89].

The FDA is currently reviewing CyclASol^®^ 0.1% cyclosporine A in F4H5 (EyeSol^®^) [90]. F4H5 extends contact time with the ocular surface to increase bioavailability and allows for a smaller drop size compared to water-based products due to low surface tension [91].

Azithromycin (AZM), a lipophilic macrolide antibiotic, inhibits bacterial growth and has secondary anti-inflammatory properties by blocking the activation of proinflammatory cytokines in corneal epithelial cells [92]. AZM is approved for ocular use in the United States and Europe in different formulations. For the 1% aqueous ophthalmic solution of AZM (AzaSite^®^) approved in the United States [93], there are some concerns about ocular surface toxicity due to excipients in the formulation [94,95]. Preservative-free medium-chain triglyceride (MCT)-based eye drops containing 1.5% AZM (Azyter^®^) have been approved in Europe [96], but mild-to-moderate discomfort occurs when instilling the drops with this formulation [97], possibly due to the MCT carrier [98,99].

Therefore, a preservative-free AZM suspension based on SFA F6H8 was developed as a non-aqueous vehicle for topical application, and its efficacy was tested in in vitro and ex vivo studies with bioluminescent bacteria [100]. The three formulations were compared in terms of their antibacterial efficacy.

No significant differences were found between the antibacterial potential of SFA-AZM, Azyter and AzaSite on the cornea. In the conjunctiva, a significant reduction in the area and intensity of bacterial bioluminescence was observed after the application of SFA-AZM, and the antibacterial efficacy of SFA-AZM was similar to that of AzaSite but better than that of Azyter [100].

The authors conclude that the preservative-free, nonaqueous SFA-based suspensions have a high potential to enhance drug uptake into the eye, especially into the conjunctiva, because the suspended drug particles adhere to the conjunctival surface [100]. SFA-AZMs could therefore be a safe and effective treatment option for ocular surface infections. However, in the future, parameters such as particle size, stability of the suspension, production costs and patient acceptance must be considered when formulating SFA-based suspensions [100].

### 5.2. SFAs for Topical Drug Delivery on the Skin

The administration of medicinal products into and through the skin is a challenging task. Since most dermally applied drugs do not penetrate the skin very well due to their physicochemical properties, high priority must be given to the selection of a suitable carrier [101]. The delivery of medicines via nanosystems into the skin has become increasingly important in recent years [102].

Hardung et al. investigated the topical application of the SFA perfluorohexyloctane (F6H8) to pig skin derived from the ear and found in that model, that the excipient itself did not penetrate the stratum corneum. However, in the same pig skin model, F6H8 was observed to increase the permeation of drugs such as diclofenac through the skin. The drug was applied as an occlusive film following topical application of the drug to the pig skin in an ethanol/water solution [30].

In a recent study characterizing nanoemulsions consisting of 20% or 40% perfluorohexyloctane (F6H8) as disperse phase loaded with the active pharmaceutical ingredient (API) diclofenac sodium and stabilized with soya lecithin (S75), a promising penetration behavior was shown in the tape stripping experiments investigated [29]. The total percentage of diclofenac sodium penetrated was about ten-times higher than that of F6H8, regardless of the formulation. In the subsequent 19F-NMR analysis, F6H8 penetrated the skin only in extremely small amounts. In summary, the results of the skin penetration experiments indicate a correlation between the skin penetration of the model drug and the disperse phase (F6H8). The authors concluded that this enhancement effect can be explained by an increasing occlusion effect with increasing SFA content of the nanoemulsions and thus the promotion of penetration into the skin. To test the assumption that the semifluorinated alkane F6H8 has an occlusion potential that increases the skin penetration of the simultaneously quantified active ingredient diclofenac sodium, in vitro occlusion studies were performed. Interestingly, the authors observed a high occlusion factor for F6H8 which reached values between approx. 83% after 1 h and 72% after 2 h. In comparison to the pure semi-fluorinated alkane, the nanoemulsion formulations (20% versus 40% SFA-content) showed moderate occlusion properties with values between 10 and 30%. The occlusion factor of the formulations with 40% SFA content was consistently higher than that of the corresponding formulations with 20%; this trend was evident for both the empty and drug-loaded formulations. These results show that the occlusive potential of the formulations depends on their F6H8 content. Occlusion reduces the evaporation of water from the skin and, thus, increases skin hydration. Skin penetration of applied substances depends on skin moisture; therefore, occlusion promotes penetration of active substances into the skin [103].

A randomized, blinded, vehicle-controlled phase 1b/2a study recently started to evaluate the safety, systemic absorption, pharmacodynamics and efficacy profile of two dose levels of a prostaglandin F2α analog dissolved in SFA and its vehicle over a six-month treatment period and compared it to a branded product containing the active ingredient minoxidil in men with androgenic alopecia. This is to facilitate the targeted delivery of the prostaglandin F2α analog in an SFA-based formulation into hair follicles [104].

Interestingly, the solubility of propofol in SFAs is very high (>300 mg/mL). Principally, almost all marketed propofol formulations are limited to intravenous application and therefore remain invasive. This makes propofol unavailable for possible non-invasive applications, such as the ambulant treatment of nausea and vomiting or refractory migraine. A non-invasive propofol administration could increase its use apart from sedation and anesthesia. SFAs might be used as excipients for propofol drug delivery by an alternative (buccal) route [105]. After buccal application of propofol diluted in the SFA perfluorobutylpentane (F4H5) examination of plasma concentrations of propofol in rats and mini-pigs followed. Rat buccal mucosa is highly keratinized in contrast to the non-keratinized pig buccal mucosa, which shows penetration behavior similar to humans [106]. Since SFAs seem to increase permeation of drugs in (keratinized) skin [29,30], this in vivo study included both rats and minipigs to emphasize the potential different extents of keratinization. Surprisingly, F4H5/propofol reached high systemic bioavailability after buccal administration in both species. The absorption was good in both anesthetized and conscious rats and mini-pigs, though the highest absorption was found in the anesthetized animals [105]. Oromucosal/buccal administration of propofol could be a noninvasive way to deliver propofol systemically and achieve high bioavailability when administered dissolved in F4H5.

### 5.3. Oral Bioavailability

Holm et al. studied F6H8 as a novel excipient in lipid-based oral drug delivery, but concluded that the applicability of F6H8 as an excipient in lipid-based formulations for poorly water-soluble drugs is very limited [107].

### 5.4. Intravenous Drug Delivery

Krafft and Riess demonstrated that SFAs stabilize PFC emulsions as co-surfactants in blood substitutes [24,45,108]. SFAs alone can form a stable emulsion and could also be used as oxygen-carriers instead of PFCs [8]. These emulsions show no toxic side effects and are nearly lipid-free. Since propofol is highly soluble in SFAs, an F6H8-based emulsion containing propofol was evaluated as a new drug-delivery system for intravenous administration. This new F6H8/propofol emulsion was compared with a commercially available lipid-based propofol emulsion (Disoprivan^®^ 1%) with respect to pharmacokinetic profile, sedation potential, clinical chemistry and histology in rats [27]. As stated above (biocompatibility section), no adverse effects were detected. The results obtained indicate that the pharmacokinetics and anesthetic properties of the two evaluated emulsions were very similar and suggest that a new developed emulsion based on the semifluorinated alkane perfluorohexyloctane (F6H8) as the disperse phase may be capable for intravenous drug delivery.

### 5.5. Pulmonary Drug Delivery

The delivery of drugs through fluorinated colloids has been studied extensively [21]. Many different drugs, such as antibiotics, corticosteroids and antitumor agents have been combined with apolar HC-in-FC emulsions. Those emulsions were balanced by FnHm diblocks acting as surfactant [24,50]. A reverse water-in-FC emulsion formulation for pulmonary drug delivery was developed which could also be used with metered-dose inhalers. Because of its positive spreading coefficient, the SFA F8H2 was chosen to facilitate dispersion over the surface of the pulmonary membrane [109].

SFAs were applied as additional components of phospholipids’ liposomes, which are often applied as drug carriers [21,53,110]. The application of SFAs makes such liposomes less permeable and more resistant to phospholipases, which generally enables the targeting of such liposomes into the required parts of the organism [53].

PFCs as drug-delivery systems are attractive—especially for use in the lungs. However, due to their hydrophobicity and lipophobicity, there is a major limitation to their use as drug carriers: the poor solubility of typical drug molecules in PFCs, resulting in formulation instabilities [111]. As stated above, SFAs have the potential to dissolve several lipophilic or water-insoluble substances [23]. Drug-delivery aspects of SFAs include their application as additional components of phospholipids’ liposomes, which are applied as drug carriers [21,53,110].

To elucidate their potential as new excipients for inhalative liquid drug carrier systems physicochemical properties of four different SFAs were evaluated [26]. In this study, two of the SFAs were suitable for aerosolization. Perfluorobutylpentane (F4H5) and perfluorohexyloctane (F6H8) presented comparable aerosolization characteristics and lipophilicity, and were therefore tested in the in vivo model in healthy rabbits. Based on the physicochemical results, the authors hypothesized that, despite very similar aerosol characteristics compared to F6H8, F4H5 would be eliminated faster because of a high vapor pressure and a low viscosity. Examination of the influence of F4H5 and F6H8 on oxygenation, carbon dioxide removal and pulmonary mechanics in healthy rabbit lungs confirmed this hypothesis. Different doses were aerosolized to test their respective effects in healthy rabbit lungs after nebulization. Interestingly, the high-dose SFA groups did not differ to a high-dose group of aerosolized saline. In comparison to untreated normal lungs (sham) a low-dose application of F4H5 showed no influence on tested parameters. The authors concluded that perfluorobutylpentane (F4H5) in a low-dose application may be suitable as a new inhalable excipient in SFA-based pulmonary drug-delivery systems for lipophilic or water-insoluble drugs.

In another study, the physicochemical properties of five different SFA/ibuprofen formulations were tested to demonstrate their ability to aerosolize and to assess their pharmacokinetics [25]. The formulations consisted of F6H8 or F4H5 and ibuprofen with or without ethanol (EtOH). All formulations were nebulizable. There were no differences in aerodynamic diameters (MMAD) between SFA and SFA/EtOH. The formulation of F4H5/EtOH-ibuprofen had the highest plasma concentration–time curve (AUC). In contrast, F6H8/EtOH had the highest deposition of ibuprofen in lung tissue but the lowest AUC. The authors concluded that the tested SFAs with ethanol as co-solvent are suitable for nebulization and pulmonary drug delivery. In particular, the F4H5/EtOH formulation could be used when rapid systemic availability is required. In this regard, the F6H8/EtOH formulation showed intrapulmonary deposition of the test substance.

### 5.6. SFA as Drug Carriers in Acute Respiratory Distress Syndrome (ARDS)

Exploring new techniques for drug delivery in ARDS lungs is of particular importance because they are characterized as normally ventilated, but also hyperinflated, poorly ventilated, and nonventilated areas. The latter can hardly be reached by a conventional aerosol [112]. It has already been shown in liquid ventilation that both PFCs and SFAs can reduce alveolar surface tension, remove alveolar exudates from dependent lung regions and allow gas exchange [11,113]. A recent study hypothesized that an aerosol with these properties could significantly increase the likelihood of effective transport and deposition of drugs in the dependent regions of the ARDS lung [112]. Since the aerosolization of SFAs and their feasibility as drug carriers were only conducted in healthy lungs, this was an interesting question. Therefore, it was investigated in an experimental animal model of ARDS whether an aerosol composed of SFAs (in this case perfluorooctylhexane; F6H8) with and without Ibuprofen had the potential to transport and deposit this lipophilic drug into both dependent and non-dependent lung regions [112]. To exclude negative side effects of the carrier, lung mechanics were followed up and documented throughout the observation period. Plasma and lung tissue ibuprofen concentrations were measured, as well as concentrations of inflammatory mediators in the lung fluid, such as TNF-α, IL-8 and IL-6.

In this regard, F6H8 appears to be a suitable drug carrier in ARDS lungs. Lung tissue samples from the most dependent parts of the lung revealed that F6H8-ibuprofen obviously reached poorly ventilated or non-ventilated lung regions. The applied dose resulted in a detectable ibuprofen plasma concentration, indicating that adequate amounts of the drug passed the alveolar capillary membrane despite damage and reached the pulmonary vasculature. Furthermore, the F6H8 aerosol alone appeared to exert anti-inflammatory effects, which was even more pronounced when F6H8-ibuprofen was administered. No negative side effects on lung mechanics were observed.

Basically, at this point, all studies evaluating SFAs as drug-delivery carriers confirmed that SFAs could be used as drug carriers in future lung applications [11,25,26,112].

### 5.7. SFAs for Protein Drug Delivery

Another promising application for SFAs is suspensions, which have been described for protein transport. Their low viscosity makes them promising as carriers for protein-powder suspensions. Suspensions containing SFAs as vehicles can benefit from their exceptional physicochemical properties, such as the low intermolecular attractive forces that lead to exceptional wettability and the inert character that limits undesirable reactions during storage [114]. Previous studies indicated high compatibility of SFAs with protein drugs [114,115].

Semifluorinated alkanes allow formulations with high concentrations of up to 280 mg/mL of monoclonal antibodies (mAb) at a low viscosity of less than 10 mPa/s and low injection forces [116].

In particular, at high mAb concentrations above 200 mg/mL, suspensions with the two tested SFAs F4H5 and F6H8 were superior to aqueous solutions in terms of viscosity and sliding force. The lowest sliding forces through a cannula were obtained with F4H5 of 8.3 ± 0.4 N compared to 40.8 ± 0.2 N for a corresponding aqueous solution at 280 mg/mL mAb. Conventional non-aqueous vehicles for pharmaceutical suspensions such as medium chain triglycerides (MCT) and sesame oil (SO) exhibited high viscosities even at low powder concentrations.

The selection of a suitable suspension vehicle for pharmaceutical high-concentration protein powder suspensions is of paramount importance. In brief, SFAs are promising due to their low viscosity, low slip forces at high concentrations and flocculent sedimentation behavior. The overall results of this study demonstrate the high potential of protein powder suspensions in these non-aqueous vehicles [116].

Another research group experimentally investigated the effect of vascular endothelial growth factor (VEGF) TrapR1R2 suspended in the SFA perfluorohexyloctane (Trap/F6H8) on corneal neovascularization [117]. Trap/F6H8 showed at least comparable anti-heme and anti-lymphangiogenesis effects in the cornea compared with the aqueous commercial VEGF TrapR1R2 (Trap) formulation. Treatment with Trap/F6H8 reduced corneal leukocyte infiltration. Trap/F6H8 does not affect corneal re-epithelialization, indicating that Trap/F6H8 is non-toxic and safe when applied to the ocular surface [117]. Administration of Trap/F6H8 as eye drops strongly inhibited corneal heme and lymphangiogenesis after 14 days of treatment in mice. The inhibition of hemangiogenesis after 3 days of treatment was even more pronounced in eyes treated with Trap/F6H8 than in eyes treated with Trap alone. These results show that the use of SFAs as vehicles enhances the early anti-hemangiogenic efficacy of Trap. Thus, SFAs as drug carriers appear to increase bioavailability, particularly in the early postinjury phase.

Other advantages of SFA formulations, particularly for proteins and antibodies, include ready-to-use eye drops with improved stability at room temperature and above, as well as enhanced drug uptake. The suspended particles deliver high local drug concentrations and increase the residence time on the cornea [91].

## 6. Conclusions and Perspectives

The aim of this review was to summarize both the clinically established and the clinical-experimental applications of SFAs.

SFAs have been used in ophthalmic surgery for many years. As novel drug carriers, they have recently been introduced in ophthalmology as eye drops. SFAs are also investigated in clinical trials for topical applications on the skin.

The clinical–experimental applications of SFAs are versatile and range from oxygen and drug carriers for the treatment of various diseases to the application and formulation of new protein suspensions. In the future, SFAs could open up entirely new possibilities for drug delivery and formulation.

## Figures and Tables

**Table 1:** Physicochemical properties of the semifluorinated alkanes perfluorobutylbutane (F4H4), perfluorobutylpentane (F4H5), perfluorobutylhexane (F4H6), perfluorobutyloctane (F4H8), perfluorohexylhexane (F6H6), perfluorohexyloctane (F6H8), perfluorohexyldodecane (F6H12) and the perfluorocarbons perfluorodecaline (PFD), perfluorooctane (PFO), perfluooctylbromide (PFOB) and water (H_2_O). CST: Critical solution temperature in olive oil (20 °C); CSTH: Critical solution temperature in hexane of selected semifluorinated alkanes (SFA) and Perfluorocarbons (PFC); adapted from Tsagogiorgas et al. [26], Le and Weers et al. [48] and Liang [49].

Substance	Molecular Weight [g/mol]	Density [g/cm^3^]	Boiling Point [°C]	Viscosity [mPas] at 25 °C	Vapor Pressure [Torr]	CSTH [°C]	CST in Olive Oil [°C]	Surface Tension [mN/m]	Interface Tension [mN/m]
F4H4							114		
F4H5	290	1.284	134	1.05	25.1 (37 °C)	<−30	68	17.43	43.0
F4H6	304	1.26 (20 °C)	124				45.1		
F4H8							<0		
F6H6	404	1.386	187	2.38	1.85 (37 °C)		121	20.0	49.6
F6H8	432	1.331	223	3.44	<1 (25 °C)	−31	70	19.65	45.3
F6H12	488	1.25	290	6.99			14	21.1	
PFD	462	1.930	142	5.1	12.5 (37 °C)	123		19	57.8
PFO	438	1.760	105	1.4	57.0 (37 °C)	158		14	55.0
PFOB	499	1.930	143	1.93	10.5 (37 °C)	68		18	51.3
H_2_O	18	0.970	100	0.89	46.9 (37 °C)	---		72	n/a

**Table 2:** Oxygen (O_2_) and carbon dioxide (CO_2_) solubilities of selected SFA and PFC adapted from Krafft and Meinert [8,24]; *^a^* from molecular simulations, given in molar fractions.

Compound	Solubility % (vol/vol)	
	O_2_	CO_2_
**SFA**		
F6H2	46.1	
	3.6 × 10^−3^ (27 °C) *^a^*	17 × 10^−3 *a*^
F6H4	44.8	
F6H6	43.4	
	4.0 × 10^−3^ (27 °C) *^a^*	22 × 10^−3 *a*^
F6H8	40.3	
F6H10	35.0	
F8H2	45.6	
F8H8	52.2 (28 °C)	
F10H2	43.4	
**PFC**		
C_8_F_17_Br (Perfluoroyctyl-bromide)	52	210
F-decalin	41.1–43	140–145

## Data Availability

No new data were created or analyzed in this study.

## Abbreviations

AKI	Acute kidney injury
ALI	Acute lung injury
ARDS	Acute Respiratory Distress Syndrome
AUC	Area under the curve
AZM	Azithromycin
CSTH	Critical temperature of solution in hexane
CST	Critical temperature of solution
DED	Dry eye disease
F4H5	Perfluorobutylpentane
F6H8	Perfluorohexyloctane
F6H8-cEM	Perfluorohexyloctane-containing emulsions
F6H8S5	perfluorohexyloctane/polydimethylsiloxane
F6H10	Perfluorohexyldecane
HBO	Hyperbaric hyperoxygenation
H_2_O	Water
H&E	Hematoxylin and eosin (staining)
mAb	Monoclonal antibodies
MCT	Medium-Chain Triglyceride
MGD	Myoboma gland dysfunction
MMAD	Mass Median Aerodynamic Diameter
mPas	Millipascal
MRT	Magnetic resonance tomography
19F-MRI	19F magnetic resonance imaging
NBO	Normobaric hyperoxygenation
NMR	Nuclear magnetic resonance
19F-NMR	19F nuclear magnetic resonance
PET	Positron emission tomography
PFCs	Perfluorcarbons
PFCL	Perfluorocarbon liquids
PFD	Perfluorodecaline
PFO	Perfluorooctane
PFOB	Perfluoroctylbromide
PLV	Partial liquid ventilation
PTFE	Polytetrafluoroethylene
PVR	Proliferative vitreoretinopathy
RES	Reticuloendothelial system
SFAs	Semifluorinated alkanes
S75	Lecithin
SH	Sodium hyaluronate
TLV	Total liquid ventilation
VEGF	Vascular endothelial growth factor
